# Supplementary material for: Divergent Evolution of Eukaryotic CC- and A-Adding Enzymes
Source: Int J Mol Sci. 2020 Jan 10;21(2):462. doi: 10.3390/ijms21020462 (PMC7014341; doi:10.3390/ijms21020462)
Supplement: Supplementary file 1 [file ijms-21-00462-s001.pdf]

Supplementary Data

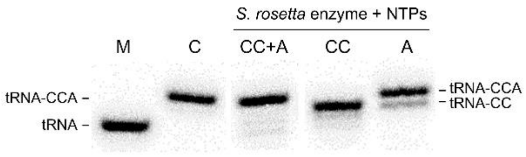

Figure S1: *S. rosetta* CC- and A-adding enzymes collaborate in CCA-addition. Equimolar amounts of *S. rosetta* CC- and A-enzyme were pooled and incubated with tRNA^{Phe} without CCA-end in the presence of NTPs. As a control, both enzymes were also incubated separately. The A-adding enzyme was incubated with tRNA^{Phe} carrying a CC-end, the CC-adding enzyme and the pooled enzymes were incubated with a tRNA^{Phe} without CCA-end. The complete CCA-sequence is only added when both enzymes are present. M, mock incubation of tRNA^{Phe} without enzymes. C, control lane with tRNA^{Phe} with CCA-end as size standard.

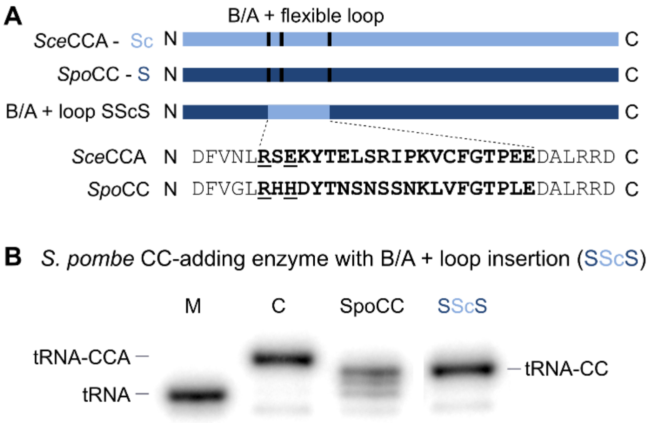

Figure S2: The flexible loop of the *S. cerevisiae* CCA-adding enzyme is not compatible with the *S. pombe* CC-adding enzyme. (A) In the CC-adding enzyme (SpoCC), both the flexible loop and the upstream located B/A motif deviate from the consensus sequence of eukaryotic e-type CCA-adding enzymes. For construction of enzyme chimeras, the B/A motif and the loop sequence from the CCA-adding enzyme of *S. cerevisiae* (Sc) were inserted into the CC-adding enzyme of *S. pombe* (S), generating chimera SScS_B/A + loop (S, S. pombe CC-adding enzyme N-terminus; Sc, B/A motif and loop of *S. cerevisiae* CCA-adding enzyme; S, *S. pombe* CC-adding enzyme C-terminus). (B) Insertion of B/A motif and loop of *S. cerevisiae* CCA-adding enzyme does not result in a restoration of A-adding activity. Obviously, this region is not compatible with the context of the *S. pombe* CC-adding enzyme due to their distant evolutionary relation. M, mock incubation of the tRNA in the absence of enzymes; C, control tRNA^{Phe} with CCA end as size marker.

Protein Sequences of CC- and A-adding Enzymes of *Salpingoeca rosetta, Schizosaccharomyces pombe*

>euhChoSalros_aNtr *Salpingoeca_rosetta* EGD81016
MLLGRVACAAVLGGVSGGGCCARSVSCCCCCWSVGAATRGRPWRRPVLQLTRRNLQPTMPTSQEVKQKILGTPAV
QQLKHIFQEDHHEFRLVGGAVRDILLERWPKDYDFATTALPQQTQQLLESRGVRVVLTGLQHGTVTAVIDNVPYE
VTTLRLDHEGAEGTGPVCFTDDWKLDAERRDLTINAMSMDLDGHIFDYFDGRDHLAQEKIVFVGDPAQRIQEDFR
ILRYFRFHGKVCRHNNHEPAQIQAITDNVQGLESVSGERIWMEMSKILKTARAPELVECMQACGVLPHIQLANVE
QHHLQRLRHVHRYELEPATALTALVDDVQQFEGVAAAWRLSNAERKLGLFIIQHRDVDVCMNSAQDLLVDGINRT
YVTQLCRYQGHLDIAVAMQDWAVPEFPITGKKLIQHGLKPGPNMGRVLAALKDNWKQSRFQLGEEELLAQMDSVV
ASLS

>euhChoSalros_eNtr *Salpingoeca_rosetta* EGD77004
MSARAHAVIEQAAATGRIRLTDLEHRVFALLIDVVQRFHLKDTLRVAGGWVRDKLLGRESLDIDITTDSLSGSDL
ALHIEQLLKEKNEKDAARFKILPVPEQSAHLQVSTIKLYDLEIDLNALRTEVYDPESRIPKVSPGTILEDTARRD
FTINALYYNINQDAIEDFTDTGLSDLDLGLVRTPADPLRSFKEDPLRVLRAIRYTGRYGFKLEDAARHAILDPQV
KECLQTKVSRERYGIEVDKMFKADHPLACLSLLCNLGLYDVVFHSPAEYHRDPTMQMVPFPYFPVPLRMIEPRLV
MEASLEIANRANKSIVAKAQDGDHHAVQTVLLSAFFSPLWGYICSQDKKVTHRSIVYHMLRRGICLSKASAEMIC
HMLFSAQQFAEVSRQVLKLYVEKHEEATAPLPDDQQQTQQQQQQQTQQQQQQQGDVDLGDLPADVRLAIGKAMVT
AGEHWRDAIILCDVFARHFHAFVIERYLPSALLMPWIEKSGLVGCWTWRPLLSGRDVMTILDIKGPMVGRLVQRI
SDEMLLQPRMTREECEQWLR
DNVQALMAECDVDKK

>eufAscSchpom_eNtr1 *Schizosaccharomyces_pombe* NP_588119.1
MYSRIVLNDVEKKVVNLLKKTADFIESKSSSSSSLEVRLAGGWVRDKLLGLSSDDLDVTLNKVTGVDFANSIFEY
VHSLDSDSVIPYKDALGKLTVNPDQSKHLETATLSLFDLDIDFVGLRAESYDDKSRIPSVTPGTVETDALRRDFT
VNTLFFNIRTEKIEDITKRGYKDLQTKVLVTPISPLQSFLEDPLRILRGIRFASRFEFTIDPSVVSAIQDPKVCK
AFEKKVSKERVGEEIEKMLKGANAKLALQLLYSTNTYQFTFDTLPAEKEFQIPKALEATESLFQSLALTFPKLMK
LSEDEKIGLWLYVALIPWSSQTVMVKKKQFYIPAIIAKDKLKLRSTYVNQLNQCCTFNPIFDELVNDTSTKNCSS
IGSLIRQLNKSWEVVFLTSVIYSCCKTPAASVSNTFSSYKSLYDFIYDKNLQNAYNMKPLLDGKQIMKNVGVKPG
PQLKETMDNMISWQFKHPEGSVEDCVAYLQSLKI

>eufAscSchpom_eNtr2 *Schizosaccharomyces_pombe* NP_594651.2
MASSSSILELNETEKELSDIFLNVSKKIGQMDRKEPEVRFAGGWVRDKLLRIESHDIDVAIDCMSGFEFAQHLQS
YLAQQHPDWETKVIKIDANPLKSKHLETATARIMGMDIDIVNLRHHDYTNSNSSNKLVFGTPLEDALRRDATINA
LFYNLKSKTVEDFTGKGLVDLSNKIIRTPLVADETFGDDPLRAVRCIRFATKYDFNIHEETIKGLKNPELHERLR
SSISRERIGVEVDKMLKHCNTNRALKIIHSLGMFACIFGPLEIHTKKLQSKNIESLSLIPYAIDLFGYLQKKDVS
IKNLSSSSKYIFWLAIATLPWYNWSILEKSKIKILPPILIRDSLKYSKPIMSQVENFFVHYPLIMSKINVLEKEG
KLTRLGCGRLVRELGPHWRDIIDWAFFMNTLISNSDIQRLNKDEEVTWFHVLVKHIEEYGMEEAYNIQPIINGNE
ITRILGIRPGHLRKMLDDSIEWRIQNPESTKEDYIAIMLEKGTSAVVDS

Protein Sequences of newly identified tRNA Nucleotidyltransferases

Choanoflagellata:

> euhChoMonbre_a-type *Monosiga_brevicollis*
built(XP_001750467.1/NW_001865078.1:179129-181381)
MASEAIKQQIISNPGLCQLGQLFQAAHHDLRLAGGAVRDLLRGLSPKDLDLATTATPEESKAILLAAHVRVVETGLQHGTVTA
VIDGEPFEITTLRIDEQTDGRRAMVVYTDDWHLDAQRRDLTINAMFMDLHGEIHDYFNGAQDLEAHRIRFVGDADERIKEDYL
RIMRYFRFHGRIALNNEHEAAQLSAIRANAAGLEQISGERVWMELAKILETTRAPDLLRTMQDCQVLNHIGMTQVTEAHLQEL
QRVHRVCRHAPTLLATLLDTAADLNALFARWRFSLAEKRHAQFILDHRASDLTLEAAEDLLVDNTPVAHVEGLCLYQEHVDWA
RHVVAFSIPSFPINGQMLMEVGVPKGRVMGRVIQALRISWKAARFTKSADQLLEEVPAILAEAQS

> euhCorCorlim_a-type *Corallochytrium_limacisporum* Betat_2015_aCCA
MDHEDKPFGRMPDNAFXWNLLNTQIPPQLRTLADVISDEGFQLRIVGGAVRDALMGKTPKDIDLGTDGTPEEILKVLETNDYR
VIPTGLQHGTVTAVAGGVNYEITSLRIDRACDGRHAVVDFTRNWRIDAERRDLTVNAMSCTLEGDLYDYFNGYEDLKNRRCLF
VGNAEKRIREDYLRILRYFRFHARIVPDPTLPGSHDEATLKALRENRHGLNRISGERIWQEMGKILTGPAAPAELRVMYDLEL
MKEIGLDQDTSVHMDHFCRMHARSHDAIACLCSLLNSPEDIEKMKKRWKFSNSEERMGMFLVTQRNKDYSLKACKDFLVDKTP
LDFVVQWTHIKDAPEADIDILKQWPIPTFPVNGGTLKKLGAKPGPSMGEMLADLKQEWKDSDFSMDQAALEERAKERISAQQE
S

> euhIchAbewhi_a-type *Abeoforma_whisleri* Betat_2015_aCCA
ILFFFLLFWMPHSFYFKKALVSKRSILRTTTTTTNILLQTLQRTPNKHLLRISNNNNSYHTNPTFDFASIRMLKQKPIQHFHR
PSSFASNYNLSTRTPLLLKKNTPVFNTAFCQRSLATMTPNTDKIRLQGHQLDRLNEIITPELKRIEEIFKQHNFDIRLVGGVV
RDIYLNQYPKDIDLATNANPHQMIEMLNKEGIRLIETGLQHGTITAVISKQSFEITSLRIDEETDGRHAVVQYTTNWKLDAER
RDLTINAMNLDLDGYLQDYFNGRQHLEQRQIFFVGAPGPRIREDYLRILRFFRFHGRISESDCYDPDTLVAIEENRSGLTRIS
GERIWTEISKIIAGNRNVNIFKTMYLCGVCESIHLPDHNRLDTLEYVSTFTLEPISLLVSLLHDLSEFENICLTWKLANTEKN
LGVFLLNHRQEFHDLLSQQESNNQVIFKYATDMLVDGVAINFLNELLLSHGQHSIVEQLKQYNLPRFPINGKDILSQGVPKGK
AVGIAMLKLKTLWKESNYQLTAEELFNHL

> euhIchAmopar_a-type *Amoebidium_parasiticum* Betat_2015_aCCA
VHEFKQSCLESLGIQICVLSSHYRAHSYACHGACLPPALMRIQTLLARVPSSLQGGIRLYFSRCHFVGSIARSGFTLKPYEIQ
YFKGSTVCNTTALFTRPLSSARHLRPLSTHTATTMGEDGVKVDLPLLGHVLTPEVKALGQLFRENGYHIRLVGGVVRDLLMGK
MPKDTDLATDAHPDQVQAMLEKANIRCVPTGLQHGTVTAVLNKTNYEITTLRIDAETDGRHAEVEFTQDWKLDAERRDLTINA
MSLDFDGHLFDYFNGREHLENRLVLFVGNADTRIKEDYLRILRYFRFHGRISDHQTHDPDVIKALRDNAAGLRRISGERVWME
MSKILAGNNAGPLLALMYDIGVAEEIGLPLSGRQSIPLMEDVRKHSRDPITVLSALLESVLGFEELFAKWKFSGQERNLGLYI
IGNRSWEMTLKFCTDELVEGVEKAKVMELARYKGHPEIADQIKDWPVPRFPVTGKHMKDKGIKPGPHMGELLRGLKQQWKASN
YTLTAEDLVATLQA

Schizosaccharomycetes:

> eufAscProino_CCA *Protomyces_inouyei* built(genome_search,prosplign)
SLTLAADEAKLTHLLQEAHAQRAFSDPAVEIRYAGGWVRDKLVQKPSHDIDIAVSSLSGHAFAQQFAAFLAKAHPDLTTGTIA
KIQANPEKSKHLDTATARFLGLELDFVQLRTESYAESGDTRTPSITGVGTLLEDAERRDCTMNALYYNVHSEKVEDPTGRGLT
DLKRGLIQTPLPPRQTFLDDPLRVLRCVRFASQFGFEIEEGTLACLAEGEVRDALDRKVVRERIGLEVYKMLRGIDPARALKT
LQSSGLYEVVFAPLPSDVSTANFEALERAMRICREEPALMQHYDAYLDGRLWLAIALLPYRDVRMPKDKRGYQDPLACGIIRD
RLKLTNALELLCRQLFPVSDTASVTADASRLELGQLVRKLGRDWRLVLLVETLASQEARSIADLKKLLERIKQEGLEDAHTFK
TPVDGKKIRALLLAAKQDIKLMSVLT

> eufAscTapdef_CCA *Taphrina_deformans* built(genome_search,prosplign)
MKRSASGSPITLSASMTTKSPQIILEPAEEQLTNLLREASSTAAFSDREVIVRYAGGWVRDKILRKKSHDIDIAISSISGHQF
AVEFAAFLASQHPDLKTGTITKILANPEKSKHLDTATSRFLNLDLDFVQLRTESYGSPDSRTPSVVDVGTLEEDAERRDCTMN
ALYYNIHTLQVEDPTSHGLDDLANGLIQTPLPPEKTFLDDPLRVLRCIRFASQFDFTIEPDTFAGMATREVRDALKCKVVKER
VGIEVYKMMKGINPAKAIRALHSQQLYQIVFQDAQPDDAFPWFDFTVVERAIDVLSKSAGLRRHLSEYSTDGRLWFIVAILPY
CRKLCPVPKKKDVEEPLGCLMVRDHLKLTSLLEALVRATFPPRQVPVTNESSPLELGKFVRLLRKDWKLAVFVAFVADEPGPR
DVDSLVALFDRIADLGLDRAWSFASFIDGKKIRPLLEKHETNIKVMKELVELVIEYRIEDPAVTEEEAMTKLRAHLETRPVGP
EG

> eufAscTapfla_CCA *Taphrina_flavorubra* built(genome_search,ncbi_genomes)
IILESAERQLTDLLREAASTSAFSDPQVVVRYAGGWVRDKILRKKSHDIDIAISSISGHQFAVEFAAFLTSQHPDLKTGTITK
ILANPEKSKHLDTATSRFLNLDLDFVQLRTESYGSADSRTPSVVGVGTLEEDAERRDCTMNALYYNVHSLEVEDPTGRGLDDL
ACGLIQTPLPPTKTFLDDPLRVLRCIRFASQFDFDIEADTLAGMATPEVRAALGCKVVKERVGIEVYKMMKGINPAKAIRTLY
DRGLYHIVFQDARPDDVISPWFSFDIVERAIDVLQRNAALRAHLNEYTEDDDGRLWFIVSLLPYCRQERPVPNKKAKGLEEPL
ACVTVRDHLKLTGLLELLVRATFPPRPVTVSLDSSPLELGRFVRSLKKDWKLALFVSFVAAADQEGDGDVSRNAAGVVDGEGL
VALMDRIATLKLDRAWSFTSFIDGGKIRPLLQEHGTHIKVMKEL

> eufAscTappop_CCA *Taphrino_populina* built(genome_search,prospign)
MKRSCSGSPIMSTPEHPTSSTKPYIGLTDEESQLTTLLREACSKAAFSDPNVVVRYAGGWVRDKILQKQSHDIDIVVSSLSGH
TFAVEFAAYLASQHPDLKQGTVSKILANPEKSKHLDTATSKFLSLDLDFVQLRTEEYGSLDSRTPDVVGVGTLQQDAERRDCT
MNALYYNIHDMAVEDPTEQGLYDLQQGLLKTPLAPKKTFLDDPLRMLRCIRFTSQFGFNIDEATFAEMKELDVRRALKEKVVK
ERVGIEVWKMMKGVDPERAIRALYSQGLYHTVFMDLEPDAVVPWFDFSIVEHAFKILKESNALKAHLIDYTDGRLCLIIAMLP
YRFEMAEVPKKPGVLEPLGCVMIRNTLKLTGALETLVRAVFPPREVTLNKDSSALELGKMVRVLKKDWKLALFVSHLSPESTM
TIEELIELMDQITALGLDTAWSFMPFIDGKKIRPLLQESKAHIK

> eufAscTapwie_CCA *Taphrina_wiesneri* built(genome_search,prospign)
MKRSASGSPITFTSSMTTKSPQIILDPAEQQLTSLLREAASTSAFSDSQVVVRYAGGWVRDKILHKKSHDIDIAISSISGHQF
AVEFAAFLTSQHPDLKTGTITKILANPEKSKHLDTATSRFLNLDLDFVQLRTESYGSADSRTPSVVDVGTLEEDAERRDCTMN
ALYYNIHTLQVEDPTRHGLDDLANGLIQTPLPPEKTFLDDPLRVLRCIRFASQFDFSIQPDTFAGMATKEVREALKCKVVKER
VGIEVYKMMKGINPPKAIRALHSQQLYQIVFQDAQPEEEFPWFDFAIVERAINVLSTSSRLRDHLSEYTADGRLWFIVAILPY
CRQLRPVPKKKDVEEPLGCVMVRDFLKLTSLLEALVRATFPPREVSVSINSTPLELGKFVRLLKKDWKLALFVSFVADSTHAV
DVDGLVALMDRITECGLDRAWSFTSFIDGKKIKPLLEEHGTHIKVMKELVELVIEYRIEDPDITEEQAMAKLREYLKTRPIAQ

> eufAscNeoirr_CCA *Neolecta_irregularis* built(genome_search,prospign)
SLSFKMTCCPEIVLTPKEERLRKALVATADHIKIKEGKDVTVRLAGGWVRDKLLSIQCNDLDVTLDSQTGYQFALKMKNYLDT
TGSSDLTSITKIESNPAKSKHLETATTRFLDLDLDFVNLRSEEYTTTSRIPEMKFGTPKEDASRRDCTINSMFYNVHTRKVED
YTXXXXXXXXXXXXXXXXXSLVKQALKLKISRERVGEEVNKMLKGPDPEYALRMICEYGLYDSVFSTWQTSIDPTLASSA
AKIVKWILESKPLSSLQLDSVSQRRLFLVAAVLPWLHQPSPDQKHIAAVVSVRDALKLPTIDSDFIKHCFMHMQDVHKAASRN
YSSENGLSRLETGLLIRVLGKNWPLIVNSSFIYDLAHTTDSSLETLKPLINGKKLANLLHLQPGPEIKNALDNLLQWQLENPD
GTATEYENLST

Loop sequences for e-type nucleotidyltransferase Weblogo generation

>Q94K06_Arabidopsis_thaliana
DFVNLRSEEYTENSRIPTMKFGTAKDDAFRRD

>C1E3J8_Micromonas_sp
DLVNLRSEEYTEASRIPEMKFGSATDDAYRRD

>A8J6P4_Chlamydomonas_reinhardtii
DLVNLRSETYAADSRIPTMTFGTPQQDALRRD

>Q42867_Lupinus_albus
DFVNLRSEEYTDNSRIPSMQRFGTPEEDAYRRD

>Q2QX33_Oryza_sativa_subsp_japonica
DFVNLRSEKYAENSRIPTVEIGTAKEDAFRRD

>A4RUG6_Ostreococcus_lucimarinus
DLVNLRSENYSEDSRIPEMQFGTAKDDAFRRD

>Q01CL7_Ostreococcus_tauri
DLVNLRSENYSEDSRIPDMEFGTAKDDAFRRD

>A9RJR2_Physcomitrella_patens
DFVNLRAETYANNSRIPTMEFGTAEQDAFRRD

>A7QGJ5_Vitis_vinifera
DFVNLRSEDYSENSRIPTMRFGTAKEDAYRRD

>B9MW94_Populus_trichocarpa
DFVNLRSEDYSENSRIPTMTFGTAKEDAYRRD

>B9T6L0_Ricinus_communis
DFVNLRCEDYTEDSRIPTMKFGTAEEDAFRRD

>Q74ZT0_Ashbya_gossypii
DFVNLRSETYAEESRVPTVEFGTPLQDAMRRD

>Q9P4S5_Candida_glabrata
DFVNLRSEEYTEDSRIPTTQFGTPEEDALRRD

>Q6BP89_Debaryomyces_hansenii
DFVNLRSEEYTTDSRVPIIECGTAEEDALRRD

>Q6CSU9_Kluyveromyces_lactis
DFVNLRSEEYTMESRIPKVEFGTPYDDAMRRD

>A7TSN1_Vanderwaltozyma_polyspora
DFVNLRSEEYTENSRIPTVEFGTPEQDALRRD

>A3LSZ1_Pichia_stipitis
DFVNLRSEEYSDDSRVPIIKFGTPEEDAYRRD

>Q2H0X0_Chaetomium_globosum
DFVNLRRETYTESSRNPVVEFGTAEEDALRRD

>A1C7Z8_Aspergillus_clavatus
DLVNLRKETYTDESRNPQMEFGTAEEDAMRRD

>A6SEN3_Botryotinia_fuckeliana
DFVNLRKETYTEDSRNPEMEFGTAEEDALRRD

```
>A7ESI2_Sclerotinia_sclerotiorum
DFVNLRKETYTEDSRNPQMEPGTAEEDALRRD

>Q8SS78_Encephalitozoon_cuniculi
DFVNLRNETYSETRIPNVKPGTPQEDAFRRD

>A9CRH2_Enterocytozoon_bieneusi
DFVHLRTESYTTSRIPQITHGTPQEDAYRRD

>Q55XT2_Cryptococcus_neoformans
DFVGLRSETYADSRIPQVKPGTPFEDASRRD

>A8Q3K4_Malassezia_globosa
DFVNLRKEVYEGTHRIPVMSFGTPLDDAMRRD

>P21269_Saccharomyces_cerevisiae
DFVNLRSEKYTELSRIPKVCFGTPEEDALRRD

>Q6CAF4_Yarrowia_lipolytica
DFVNLRSEEYSDESRVPVVQFGTAEQDAYRRD

>A4RHL3_Magnaporthe_grisea
DFVNLRKETYTEDSRNPQMEFGTAEEDALRRD

>Q7SAL9_Neurospora_crassa
DLVNLRKETYTEDSRHPQVEFGTAEEDALRRD

>A1DIN0_Neosartorya_fischeri
DLVNLRKETYTDDSRNPQMEYGTAEEDALRRD

>Q4WZK0_Aspergillus_fumigatus
DLVNLRKEMYTDDSRNPQMEFGTAEEDALRRD

>A2QJY3_Aspergillus_niger
DLVNLRKETYSEDSRNPQMEFGTAQEDAMRRD

>Q2UB09_Aspergillus_oryzae
DLVNLRKETYSDDSRNPQMEFGTAEEDAMRRD

>Q0CVM0_Aspergillus_terreus
DLVNLRKETYTDDSRNPQMEFGTAEEDAMRRD

>Q1DJQ5_Coccidioides_immitis
DLVNLRKETYSDHSRHPEMEFGTAEEDALRRD

>Q5AWL8_Emericella_nidulans
DLVNLRKEAYDENSRTPQMEFGTAEEDALRRD

>A6R867_Ajellomyces_capsulata
DLVNLRKETYSDDSRNPQVEIGTPEEDALRRD

>Q0UH04_Phaeosphaeria_nodorum
DLVNLRKETYNEVSRNPQMEFGTAEEDAMRRD

>C4V7M3_Nosema_ceranae
DFVNLRCEKYTDTRIPLITIGTAQEDAFRRD

>B0CQW9_Laccaria_bicolor
DLVNLRSEEYSETSRIPTEVLFGTPLEDARRRD

>Q4P7C9_Ustilago_maydis
DFVNLRKETYDPGSRIPTMTFGTPKEDAERRD
```

>A7AWW8_Babesia_bovis
DFVNLRSEDYAQNSRIPIMKIGTPFEDAMRRD

>Q5CPD4_Cryptosporidium_hominis
DFVGLRSETYTLESRIPIITLGTAEEDAFRRD

>Q5CUP1_Cryptosporidium_parvum
DFVGLRSETYTLESRIPIITLGTAEEDAFRRD

>Q4YRW2_Plasmodium_berghei
DIVNLRNEKYTEESRIPEISIGTVEEDALRRD

>Q4UBS4_Theileria_annulata
DFVNLRTEDYSSNTRIPDMKIGTPVEDAMRRD

>A0BX57_Paramecium_tetraurelia
DFVNLRGETYTQNSRTPQIVVGTPEQDAFRRD

>A8BT44_Giardia_lamblia
DLVNLRAESYQETSRVPSAIKFGSPHEDALRRD

>Q4Y283_Plasmodium_chabaudi
DIVNLRNETYTEESRIPEIAIGTPEEDALRRD

>Q8IIG2_Plasmodium_falciparum
DIVNLRNEKYTEESRIPEIVIGTPEEDALRRD

>Q7PDU7__Plasmodium_yoelii_yoelii
DIVNLRNEKYTEESRIPEISIGTVEEDALRRD

>A5K4L8_Plasmodium_vivax
DVVNLRNEKYTEDSRIPEIIIGTPEEDALRRD

>Q4MZK9_Theileria_parva
DFVNLRTEDYSSNTRIPDMKIGTPLEDAMRRD

Loop sequences for a-type nucleotidyltransferase Weblogo generation

```
>euhIchSphdes [Sphaerothecum_destruens_NA]
EITTLRVDSEHDGRWAQCSWTRDWREDALRRD

>euhFilMinvib [Ministeria_vibrans_NA]
EVTTLRVDTATDGRFAEVKYTSSWREDAERRD

>euhCorCorlim [Corallochytrium_limacisporum_NA]
EITSLRIDRACDGRHAVVDFTRNWRIDAERRD

>euhIchCrefra [Creolimax_fragrantissima_NA]
EITTLRIDTITDGRHAEVDFTTSWVEDAARRD

>euhMetDromel [AAF51964_Drosophila_melanogaster]
EVTTLRIDIRTDGRHAEVMYTTDWQLDANRRD

>euhIchAbewhi [Abeoforma_whisleri_NA]
EITSLRIDEETDGRHAVVQYTTNWKLDAERRD

>euhIchPirgem [Pirum_gemmata_NA]
EVTSLRIDKETDGRHAVVEWRKDAERRD

>euhMetHomsap [BAB70662_Homo_sapiens]
EITTLRIDVTTDGRHAEVEFTTDWQKDAERRD

>euhMetStrpur [XP_784774_Strongylocentrotus_purpuratus]
EITTLRIDRVTDGRHAEVEFTTDWRTDAERRD

>euhChoMonbre [EDQ84681_Monosiga_brevicollis]
EITTLRIDEQTDGRRAMVVYTDDWHLDAQRRD

>euhMetAmpque [XP_003390969_Amphimedon_queenslandica]
EITTLRIDHVTDGRHALVQFTSDWVKDAERRD

>euhMetNemvec [XP_001637889_Nematostella_vectensis]
EVTTLRIDVETDGRHAKVKFTNDWQLDAERRD

>euhMetTriadh [XP_002112522_Trichoplax_adhaerens]
EITTLRIDAITDGRHAQVQFTNDWRIDAERRD

>euhIchAmopar [Amoebidium_parasiticum]
EITTLRIDAETDGRHAEVEFTQDWKLDAERRD

>euhIchIchhof [Ichthyophonus_hoferi_NA]
EITTLRIDEVTDGRHAEVVFTQDWKLDAERRD

>euhFilCapowc [EFW46942_Capsaspora_owczarzaki]
EVTTLRVDKITDGRHAEVEFTSDWSLDAQRRD

>AniLupus[533758_anis_lupus_familiaris_(dog)]
EITTLRIDVVTDGRHAEVEFTTDWQKDAERRD

>IxoScap[B7Q4B3_Ixodes_scapularis]
EVTTLRIDVVTDGRHAEVEFTTDWETDANRRD

>BOVIN [Q1RML6_BOVIN]
EITTLRIDVATDGRHAEVEFTTDWQKDAERRD

>RatNor[Q4VBH2_RAT]
EVTTLRIDVSTDGRHAEVEFTTDWQKDAERRD
```

>MacMula[XP_001100673_1_Macaca_mulatta]
EITTLRIDVTTDGRHAEVEFTTDWQKDAERRD

>TaeGutt[XP_002186976_Taeniopygia_guttata]
EITTLRIDVVTDGRHAEVEFTTDWHKDAERRD

>MonDome[1_XP_001374161_Monodelphis_domestica]
EITTLRIDVITDGRHAEVEFTTDWQKDAERRD

>MusMusc[Q8K1J6_Mus_musculus]
EVTTLRIDVTTDGRHAEVEFTTDWQKDAERRD

>OrnAnat[XP_001506360_1_Ornithorhynchus_anatinus]
EITTLRIDVMTDGRHAEVEFTTDWEKDAGRRD

>XenTrop[Q6P873_Xenopus_tropicalis]
EITTLRVDVQTDGRHAEVEFTTDWETDAERRD

>DanReri[Q6IQR7_Danio_rerio]
EVTTLRVDVQTDGRHAEVEFTTDWQKDAERRD

>TetNigr[Q4SR02_Tetraodon_nigroviridis]
EVTTLRVDVQTDGRHAEVEFTTDWQKDAERRD

>NemVect[A7RRV1_Nematostella_vectensis]
EVTTLRIDVETDGRHAKVKFTNDWQLDAERRD

>AedAegy[Q17N51_Aedes_aegypti]
EITTLRIDAVTDGRHAEVIHTTDWLLDANRRD

>AnoGambi[Q7QCF2_Anopheles_gambiae]
EITTLRIDAITDGRHAEVIHTTDWLLDANRRD

>CulQuin[B0X1I4_Culex_quinquefasciatus]
EITTLRIDAVTDGRHAEVIHTKDWLLDANRRD

>CaeEleg[Q93795_Caenorhabditis_elegans]
EITTLRVDIVCDGRRAQVEYTTDWQLDANRRD

>TriCast[XP_969345_Tribolium_castaneum]
EVTTLRIDVVTDGRRAEVQFTTDWLLDALRRD

Loop sequences of a-type enzymes from arbitrarily chosen Choanoflagellata

>*Hartaetosiga_balthica*
EITTLRLDHSINGVAMETQCFTDDWELDALRRD

>*Salpingoeca_kvevrii*
EISTLRIDPGNLAAVDEEDSEAPWRADAACRD

>*Microstomoeca_roanoka*
EVTTLRLDEHTCEDTSKEKAPQADEQIIFTENWKLDAERRD

>*Salpingoeca_punica*
EITTLRTDENDSAPDRTFLLDAKCRD

>*Salpingoeca_rosetta*
EVTTLRLDHEGAEGTGPVCFTDDWKLDAERRD

Abbreviations used in Figure 2 and 3

Abbreviation	Organism	Accession number
bAquAquaeo_A	*Aquifex aeolicus*	NP_213288
bAquAquaeo_CC	*Aquifex aeolicus*	NP_214480
bBciBachal_A	*Bacillus halodurans*	NP_243747
bBciBachal_CC	*Bacillus halodurans*	NP_242550
bCyaSyysp_A	*Synechocystis sp.*	NP_441479
bCyaSyysp_CC	*Synechocystis_sp.*	NP_442458
bDeiDeirad_A	*Deinococcus radiodurans*	NP_294707
bDeiDeirad_CC	*Deinococcus radiodurans*	NP_294915
bDeiThethe_A	*Thermus thermophilus*	YP_144097
bDeiThethe_CC	*Thermus thermophilus*	YP_144191
bSpiBorbur_CCA	*Borrelia burgdorferi*	ZP_03086907
bTheFernod_CCAIIa	*Fervidobacterium nodosum*	A7HLG0
bTheThemar_CCAIIa	*Thermotoga maritima*	Q9WZH4
baEhrcha_CCA	*Ehrlichia chaffeensis*	Q2GF83
baRhilot_CCA	*Rhizobium loti*	Q98HS9
baRicpro_CCA	*Rickettsia prowazekii*	Q9ZEC8
bdGeosul_A	*Geobacter sulfurreducens*	NP_952632
bdGeosul_CC	*Geobacter sulfurreducens*	NP_953233
bgEsccol_CCA	*Escherichia_coli*	P06961
bgWigglo_CCA	*Wigglesworthia glossinidia*	Q8D2W4
ebChlAratha_CCA	*Arabidopsis thaliana*	Q94K06
ebChlLupalb_CCA	*Lupinus albus*	AAB03077.1
ebChlPhypat_CCA	*Physcomitrella patens*	A9RJR2
eufAscAspnid_CCA	*Aspergillus nidulans*	KZN89459
eufAscCangla_CCA	*Candida glabrata*	XP_449283.1
eufAscKlulac_CCA	*Kluyveromyces lactis*	AAG00316
eufAscNeoirr_CCA	*Neolecta irregularis*	NA
eufAscNeucra_CCA	*Neurospora crassa*	XP_962692
eufAscPnecar_CCA	*Pneumocystis carinii*	XP_018225359.1
eufAscPnejir_CCA	*Pneumocystis jirovecii*	XP_018230996.1
eufAscPnemur_CCA	*Pneumocystis murina*	XP_007874977.1
eufAscProino_CCA	*Protomyces inouyei*	NA
eufAscProlac_CCA	*Protomyces lactucaedebilis*	ORY85595
eufAscSaccer_CCA	*Sacharomyces cerevisiae*	NP_011095
eufAscSaicom_CCA	*Saitoella complicata*	XP_019025108.1
eufAscSchcry_A	*Schizosaccharomyces cryophilus*	EPY50617_Scry/1489
eufAscSchcry_CC	*Schizosaccharomyces cryophilus*	EPY50393_Scry/1538
eufAscSchjap_A	*Schizosaccharomyces japonicus*	EEB06657_Sjap/1487
eufAscSchjap_CC	*Schizosaccharomyces japonicus*	EEB07205_Sjap/1526
eufAscSchoct_A	*Schizosaccharomyces octosporus*	EPX70971_Soct/1489
eufAscSchoct_CC	*Schizosaccharomyces octosporus*	EPX73121_Soct/1536
eufAscSchpom_A	*Schizosaccharomyces pombe*	NP_588119.1
eufAscSchpom_CC	*Schizosaccharomyces pombe*	NP_594651.2
eufAscTapdef_CCA	*Taphrina deformans*	NA

eufAscTapfla_CCA	*Taphrina flavorubra*	NA
eufAscTappop_CCA	*Taphrino populina*	NA
eufAscTapwie_CCA	*Taphrina wiesneri*	NA
euhCorCorlim_a-type	*Corallochytrium limacisporum*	NA
euhFilCapowc_a-type	*Capsaspora owczarzaki*	Cowc_CAOG_04900
euhIchAbewhi_a-type	*Abeoforma whisleri*	NA
euhIchAmopar_a-type	*Amoebidium_parasiticum*	NA
euhIchCrefra_a-type	*Creolimax fragrantissima*	CFRG8205T1
euhMetCaeele_CCA	*Caenorhabditis elegans*	Q93795
euhMetDromel_CCA	*Drosophila melanogaster*	AAF51964
euhMetHomsap_CCA	*Homo sapiens*	BAB70662
euhMetStrpur_CCA	*Strongylocentrotus purpuratus*	XP_787015
euhChoAcaspe_a-type	*Acanthoeca spectabilis*	lcl|GGPA01036680.1
euhChoChoper_a-type	*Choanoeca perplexa*	lcl|GGOP01021473.1
euhChoCodhol_a-type	*Codosiga hollandica*	lcl|GGOV01001436.1
euhChoDiagra_a-type	*Diaphanoeca grandis*	lcl|GGPB01030090.1
euhChoDidcos_a-type	*Didymoeca costata*	lcl|GGOQ01024127.1
euhChoHarbal_a-type	*Hartaetosiga balthica*	lcl|GGOO01006806.1
euhChoHargra_a-type	*Hartaetosiga gracilis*	lcl|GGOU01008309.1
euhChoHelnan_a-type	*Helgoeca nana*	lcl|GGOR01029434.1
euhChoMicroa_a-type	*Microstomoeca roanoka*	lcl|GGON01002325.1
euhChoMonbre_a-type	*Monosiga brevicollis*	NA
euhChoMylflu_a-type	*Mylnosiga fluctuans*	lcl|GGOI01002178.1
euhChoSaldol_a-type	*Salpingoeca dolichothecata*	lcl|GGOK01027531.1
euhChoSalhel_a-type	*Salpingoeca helianthica*	lcl|GGOJ01023652.1
euhChoSalinf_a-type	*Salpingoeca infusionum*	lcl|GGOW01010160.1
euhChoSalkve_a-type	*Salpingoeca kvevrii*	lcl|GGOX01015633.1
euhChoSalmac_a-type	*Salpingoeca macrocollata*	lcl|GGOT01013341.1
euhChoSalpun_a-type	*Salpingoeca punica*	lcl|GGOZ01014644.1
euhChoSalros_a-type	*Salpingoeca rosetta*	EGD81016
euhChoSalurc_a-type	*Salpingoeca urceolata*	lcl|GGOY01009706.1
euhChoSavpar_a-type	*Savillea parva*	lcl|GGOL01014571.1
euhChoStedip_a-type	*Stephanoeca diplocostata*	lcl|GGOM01014527.1
euhChoChoper_e-type	*Choanoeca perplexa*	lcl|GGOP01015435.1
euhChoHarbal_e-type	*Hartaetosiga balthica*	lcl|GGOO01012516.1
euhChoHargra_e-type	*Hartaetosiga gracilis*	lcl|GGOU01003296.1
euhChoMicroa_e-type	*Microstomoeca roanoka*	lcl|GGON01033848.1
euhChoMonbre_e-type	*Monosiga brevicollis*	EDQ88852
euhChoSalinf_e-type	*Salpingoeca infusionum*	lcl|GGOW01025095.1
euhChoSalkve_e-type	*Salpingoeca kvevrii*	lcl|GGOX01014948.1
euhChoSalmac_e-type	*Salpingoeca macrocollata*	lcl|GGOT01008786.1
euhChoSalpun_e-type	*Salpingoeca punica*	lcl|GGOZ01000957.1
euhChoSalros_e-type	*Salpingoeca rosetta*	EGD77004
euhChoSalurc_e-type	*Salpingoeca urceolata*	lcl|GGOY01035099.1
